# Early Human Speciation, Brain Expansion and Dispersal Influenced by African Climate Pulses

**DOI:** 10.1371/journal.pone.0076750

**Published:** 2013-10-16

**Authors:** Susanne Shultz, Mark Maslin

**Affiliations:** 1 Faculty of Life Sciences, The University of Manchester, Manchester, United Kingdom; 2 Department of Geography, University College London, London, United Kingdom; University of Oxford, United Kingdom

## Abstract

Early human evolution is characterised by pulsed speciation and dispersal events that cannot be explained fully by global or continental paleoclimate records. We propose that the collated record of ephemeral East African Rift System (EARS) lakes could be a proxy for the regional paleoclimate conditions experienced by early hominins. Here we show that the presence of these lakes is associated with low levels of dust deposition in both West African and Mediterranean records, but is not associated with long-term global cooling and aridification of East Africa. Hominin expansion and diversification seem to be associated with climate pulses characterized by the precession-forced appearance and disappearance of deep EARS lakes. The most profound period for hominin evolution occurs at about 1.9 Ma; with the highest recorded diversity of hominin species, the appearance of *Homo (sensu stricto)* and major dispersal events out of East Africa into Eurasia. During this period, ephemeral deep-freshwater lakes appeared along the whole length of the EARS, fundamentally changing the local environment. The relationship between the local environment and hominin brain expansion is less clear. The major step-wise expansion in brain size around 1.9 Ma when *Homo* appeared was coeval with the occurrence of ephemeral deep lakes. Subsequent incremental increases in brain size are associated with dry periods with few if any lakes. Plio-Pleistocene East African climate pulses as evinced by the paleo-lake records seem, therefore, fundamental to hominin speciation, encephalisation and migration.

## Introduction

**Figure 1:**
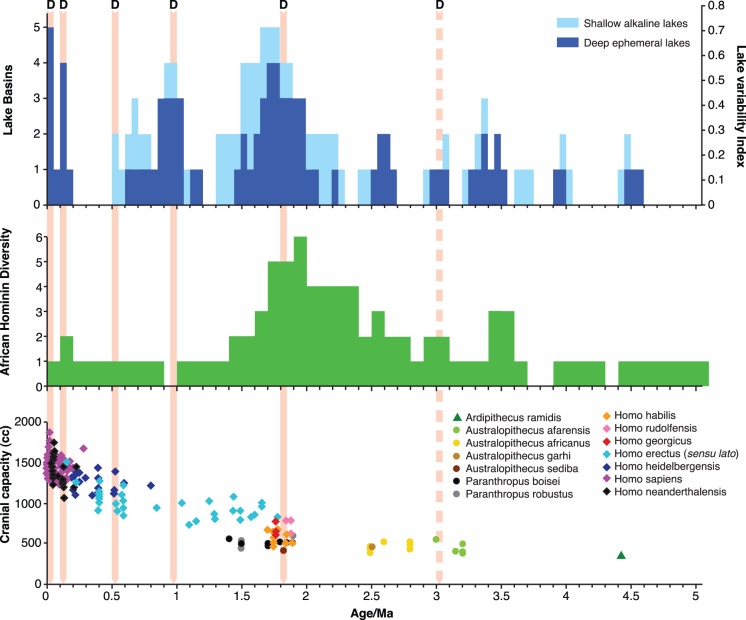
Top panel shows the East African Rift valley lake variability shown both as the number of Basin containing deep or shallow lakes and the calculated normalised lake index. The putative hominin dispersals ‘D’ (red arrows out of Africa, dotted within Africa only) are shown above. Middle panel shows African hominin species diversity over time. Bottom panel shows hominin brain estimates for Africa and Eurasia. Hominin specimen dates and brain size estimates were taken from Shultz et al [3]. East African hominin diversity at each 100 kyrs interval were estimated using first (FAD) and last appearance dates (FAD) from the literature [2], [35]–[36], [46]. *Homo erectus* and *H. ergaster* were treated as a ‘super-species’ referred to in the Figure key and text as ‘*Homo erectus (sensu lato)*’, but distinct regional processes in brain size change were identified by separating the specimens by continent in the analyses. Hominin migration dates were estimated by FAD of hominin specimens outside of EARS. Lake variability index was calculated by collating the published geological evidence for the appearance of either deep ephemeral or shallow alkaline lakes in seven major Basins [15], [17], [23]–[27], [34]–[37]. The index was normalised by dividing by 7 to produce a range from 0 to 1.

Human evolution is characterised by speciation, extinction and dispersal events that cannot currently be explained by global or regional paleoclimate records [1]–[3]. Despite the widespread distribution of hominins across Africa and Eurasia after two million years ago, the majority of hominin species originated in East Africa and subsequently colonised the rest of Africa and Eurasia [4]–[5]. Arguably the most important episode in hominin evolution occurred in this region during the period bracketing 1.8–1.9 Ma when hominin diversity reached its highest level with the appearance of the *Homo (sensu stricto)* and new species of the *Paranthropus* genera. In addition to speciation, a second major process that begins during this period is the episodic migration of hominins out of the Rift Valley and into Eurasia. This period also witnessed the most dramatic increases in hominin brain size; early representatives of the *Homo erectus sensu lato* (*Homo erectus* and *H. ergaster*) in Africa had a brain that was >80% larger than the gracile australopithecine *Australopithecus afarensis* and ∼40% larger than *Homo (Australopithecus) habilis* (Figure 1). However, there remains no consensus for what conditions or mechanisms drove these major changes.

Environmental pressures have long been assumed to play a key role in hominin speciation and adaptation [6]. The *savannah hypothesis* implicates the long-term trend towards increased aridity and the expansion of the savannah as a major driver of hominin evolution [7]–[8]. As opposed to long-term directional trends, the *variability selection hypothesis* advocates the role of environmental unpredictability in selecting for behavioural or ecological flexibility [9]–[12]. However, these hypotheses, which emphasise long-term trends toward either a drier or more variable climate, do not explain the pulsed nature of hominin speciation and migration events. Two alternative hypotheses, however, can account for the discrete and episodic nature of hominin evolutionary events. The *turnover pulse hypothesis*, developed to explain discrete patterns in ungulate speciation, suggests that acute climate shifts drive adaptation and speciation [13]. However, the evidence for this hypotheses is equivocal as the stacked benthic foraminifera δ^18^O record, which is thought to represent global climate, may [14] or may not [1] contain temporal signatures associated with key events in hominin evolution. A more recent hotly debated hypothesis, is the *pulsed climate variability hypothesis*, that highlights the role of extreme wet-dry climate cycles specific to East Africa in driving hominin evolution [15].

This last hypothesis is based on the distinct geologic and climate conditions of East Africa. The East African Rift System (EARS), one of the most extensive geological features on the Earth’s surface, runs north-south for around 4500 km from Syria through East Africa to Mozambique [16]–[17]. The formation of the EARS had a profound effect on the long-term climate of East Africa. Evidence from carbon isotope records from both soil carbonates [18]–[20] and biomarkers (*n*-alkanes) extracted from deep-sea sediments [21] provide clear evidence of a progressive vegetation shift from C3 (∼trees and shrubs) to C4 (∼tropical grasses) plants during the Plio-Pleistocene. This shift has been ascribed to increased aridity due to the orographic barrier produced by the progressive rifting of East Africa during this period [22].

The progressive formation of the EARS also led to the production of isolated basins within which lakes could form. Southward propagation of rifting and magmatic activity resulted in the formation of lake basins first in the northern parts of the EARS. For example, fluviolacustrine history of the Afar, Omo-Turkana and Baringo-Bogoria Basins in the north began in the Middle and Late Miocene, whereas the oldest lacustrine sequences in the central and southern segments of the rift in Kenya and Tanzania are found in the Early Pliocene [23]. Despite the southward progression of tectonic processes in East Africa, ephemeral deep-water lakes seem to occur in separate basins at approximately the same time suggesting a climatic control [17], [24]. Figure 1 shows a compilation of all the current known occurrences of both ephemeral deep freshwater and shallow alkaline lakes in the EARS for the last 5 Myrs. There is growing evidence that during each of these major lake phases the large fresh-water lakes appeared and disappeared on a precessional time-scale [24]–[27], which may reflect repeated periods of extremely wet and arid conditions and significant vegetation shifts within the EARS [28]. Up to and including 2.6 Ma, these lakes track 400 ka eccentricity cycles. Subsequently, these lake phases still occur at the peak eccentricity forcing but only at ∼1.8 Ma and ∼1.0 Ma. The most significant lake phase at 1.9–1.7 Ma [1], that corresponds with the intensification of the Walker circulation [29]–[31]. At this time the E-W sea surface temperature gradients in both the Pacific and Indian Oceans increased [29], [31] intensifying the E-W moisture transport in the tropics, which greatly increased rainfall variability both on a precession and an ENSO (El Niño Southern Oscillation) time-scales.

## Materials and Methods

### Data

Regional aeolian terrigenous dust records from off the coast of West Africa (ODP 659), Arabian Sea (ODP 721/722) and Mediterranean (ODP 967) and global stacked benthic foraminifera δ^18^O paleoclimate records were collated from published sources (see File S1). Data on hominin species first and last appearance dates (FAD and LAD), postulated timing of dispersal events and brain size were collated from the literature (see Tables S1 and S2 in File S1). Absolute hominin brain capacity was used with no correction for body size for two reasons. First there is significant controversy over body size estimates and second recent work shows that absolute brain size is the best predictor for cognitive ability [32]–[33]. We used the FAD and LAD to generate estimates of hominin diversity during each 100 kyrs block between the present and 3 Ma. We then derived a species turnover vector by differencing richness estimates from adjacent time periods. The lake variability index was calculated by collating the published geological evidence for the appearance of either deep ephemeral or shallow alkaline lakes in seven major Basins in 50 kyrs sections over the last 5 Myrs [15], [17], [23]–[27], [34]–[37]. These Basins are; Olduvai (Tanzania), Magadi-Natron-Olorgesailie (N. Tanzania and S. Kenya), Central Kenya Rift (Kenya), Baringo-Bogoria (Kenya), Omo-Turkana-Suguta (N. Kenya), Ethiopian Rift (South and Central Ethiopia) and Afar (N. Ethiopia). The index was normalised by dividing by 7 to produce a range from 0 to 1.

### Statistics

**Figure 2:**
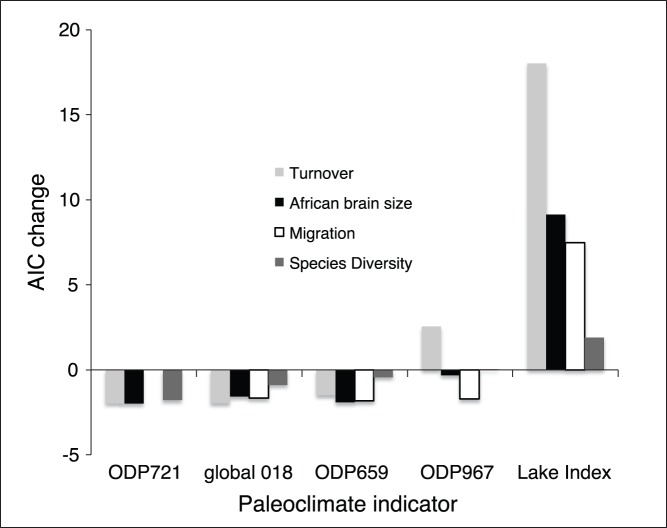
Relative impact on model fit of different paleoclimate indicators as predictors of aspects of African hominin evolution (species turnover, brain size change, dispersal events, and overall diversity). Values represent the deltaAIC change [38] from dropping each of the indicators from the global model. ODP721 was not incuded in the migration analyses due to a high VIF. Non-climate variables included in these models are not shown here as they vary across analyses but can be found in File S1.

To identify best fit models relating paleoclimate to both the lake index and hominin evolution, we used a the stepAIC function in R package MASS to select the best fit model [38], see Figure 2. For comparison of fit, we then ran these models through a linear model to extract p values and r^2^ (see File S1).

## Results and Discussion

To establish whether EARS lake formation is associated with paleoclimate trends, we used regional (aeolian terrigenous dust records from off the coast of West Africa ODP 659, Arabian Sea ODP 721/722 and Mediterranean ODP 967) and global (stacked benthic foraminifera δ^18^O) paleoclimate records to predict the occurrence of EARS lakes (see File S1). The best-fit model requires both the regional and global records to explain what is occurring within the Rift valley as evinced by the collated lake record (Table 1). As it has been suggested that the lakes represent pulsed changes in local climate conditions, we also incorporated measures of climate pulses or shifts in regional dust records [2] (Table 1). These measures of climate turnover substantially improve model fit, indicating that the occurrence of widespread ephemeral deep lakes is a pulsed system influenced both by regional and global changes. Hence over the Plio-Pleistocene, East African climate is best characterised as a long-term trend towards increasing aridity punctuated by periods of precession-forced high rainfall leading to the periodic appearance of deep freshwater lakes. It is these pulses of increased productivity and shifts in habitat availability that may explain hominin speciation and dispersal events.

**Table 1:** EARS lake activity is predicted by regional paleoclimate records.

Model	AIC	adj r2	Predictors	d.f.	AIC change	Coefficients
Regional and global paleoclimate records	−18.99	0.16	ODP659	1,56	4.09	−0.55 (0.22)
			δ^18^O Stack		3.78	1.32 (0.56)
			ODP967		3.13	−0.41 (0.18)
			ODP721/722		2.34	0.39 (0.19)
As above with additional climate variability measures	−37.48	0.49	APL ODP 967	1,39	7.58	0.07 (0.02)
			APL ODP 659		6.66	0.09 (0.03)
			δ^18^O stack		5.39	1.64 (0.63)
			ODP 967		3.79	−0.53 (0.22)
			ODP 659		3.38	−0.24 (0.11)
			ODP 721/722		0.35	0.29 (0.20)

Full model results are presented in the supplementary information. Best fit model was identified using the stepAIC function in R package Mass [38]. APL, a measure of climate pulses or turnover, represents average path length for each 50 kyrs period as derived by Donges et al. [2], AIC change represents the impact on model fit by dropping each predictor (positive change indicates that dropping a factor worsens model fit). Variables listed in decreasing order of influence on model fit. The first model covers the period from 0–3 Ma and the second from 350 Ka −2.6 Ma, due to the reduced coverage of the APL records.

As the lakes appear to be a proxy for local climate pulses, we use the lake index to explicitly test the *pulsed climate variability hypothesis* [15] by evaluating the relationship between paleoclimate and hominin evolution. Our approach is a significant advance on previous models, as we incorporate the lake index as a proxy for local climate, together with regional and global paleoclimate records. By incorporating multiple records, we can identify which aspects of paleoclimate best predict hominin evolution. Over the past 3 Myrs, East African hominin species diversity and dispersal events are predicted by both lake presence and Mediterranean dust deposition (Table 2 and Figure S1 in File S1). Of these two predictors, the lake index has the strongest impact on model fit for both the dispersal and diversity models. In the period following 2 Ma, the fossil record suggests there are periodic hominin dispersal events out of East Africa [39]. Around 1.8 Ma, there is evidence of *Homo georgicus* and *H. erectus* in Eurasia and *H. habilis* in southern Africa suggesting they may have migrated out of East Africa before this time. This first pulse is followed by later dispersals into both Eurasia and southern Africa by *H. heidelbergensis* and finally anatomically modern *H. sapiens*. Previous explanations for the onset of dispersal have been tied to hominins acquiring the necessary competencies in foraging or locomotion, life history [40], material technology or cognition to expand their range. However, the small-brain and body sizes and technologically crude early Dmanisi hominins may suggest that it was not a cognitive, a technological or a locomotory threshold, which facilitated early dispersal [41]. Additionally, once hominins overcame the constraining factors, one would assume that there would be continual, if low-level dispersal. In contrast, the fossil record suggests that dispersal events were discrete events. The regional differentiation between African and Asian *H. erectus* populations, and the eventual transition of the former into *H. heidelbergensis*, and between *H. sapiens* and *H. neanderthalensis* suggests that there was limited gene flow between Africa and Asia. A parsimonious explanation for periodic migration events is that they were forced or facilitated by climatic conditions. We suggest that both the lake presence and absence could be associated with dispersal events. For example when the lake Basins are dry they become ‘hyper arid’ and thus inhabitable and hence hominin population would have been forced to migrate to the north and south [34] but there would also have been a server lack of resources. Thus dispersal is more likely to have occurred when the Basins were completely filled with water, as there would have been limited space for the hominin populations on the tree covered Rift shoulders and river flood plains [34]. The wet conditions would however been more conducive to dispersal as hominin populations would have expanded due to the availability of water and food and could have followed the Nile tributaries northward [42]. So the occurrence of deep freshwater lakes would have forced expanding hominin populations both northwards and southwards generate a pumping effect pushing them out of East Africa towards the Ethiopian highlands and the Sinai Peninsula or into Southern Africa with each successive precessional cycle.

**Table 2:** Lake presence and Mediterranean dust records predict hominin dispersal and diversity patterns (0–3 Ma).

Model	AIC	adj r2	Predictor	AIC change	Coefficient
Migration	16.04		Deep Lakes	13.35	17.14 (7.64)
Diversity	96.50	0.49	All lakes	19.508	5.9 (1.21)
			ODP 967	6.534	−2.8 (0.95)
Species Turnover	79.11	0.18	ODP 967	2.551	2.10 (1.01)
			Deep lakes	3.986	−2.69 (1.12)
			African diversity	6.738	0.39 (0.13)

The migration model is one of three scenarios (see Table S2 in File S1). As species richness is temporally autocorrelated, we also ran a model of species turnover (net change in richness for each time period). All global and regional paleoclimate databases were included in the model. As above, StepAIC was used to identify best-fit models. No r2 is reported for migration as we assumed a binomial error structure. Full model results are available in File S1.

A final characteristic that defines hominin evolution is increasing brain size over the Pleistocene. Figure 1 shows a new compilation of estimated cranial capacity [3], with each specimen plotted against its date. Hominin encephalisation is a combination of processes: an underlying gradual trend towards larger brains is punctuated by several large step increases at ∼100 Ka and ∼1.9 Ma [3]. Global benthic foraminifera δ^18^O and Indian Ocean aeolian records do not explain these processes [3]. Here we use a comprehensive set of paleoclimate indicators: East African Rift lake presence, regional Aeolian flux records from the Arabian Sea, the Mediterranean and the East Atlantic together global benthic foraminifera δ^18^O with to develop models predicting hominin brain size. The largest change in brain size, associated with the appearance of the *Homo erectus* super-species is associated with the period of maximal ephemeral lake coverage. This jump of 80% expansion in cranial capacity occurs during one of only two periods when there is evidence for at least 5 of the 7 major intra-rift lake basins being active. Subsequently, the underlying trend towards increasing brain size in *Homo* is most strongly correlated with both decreases in lake presence and high levels of dust deposition in the Mediterranean ODP967 record (Table 3), indicating drier conditions in East Africa [43]. Brain size change in African hominins was not predicted by either Arabian Sea dust or global benthic foraminifera δ^18^O records. In contrast, patterns of hominin encephalisation in Eurasia were *positively* associated with lake presence. This later finding stems from the association between dispersal events and lakes: larger brained African hominins colonised Eurasia during periods when extensive lakes in the EARS push them out of Africa (Figure 1). Taken together, this suggests that small steps in brain expansion in Africa may have been driven by regional aridity. In contrast, the great leap forward in early *Homo* brain size at 1.8 Ma was associated with the novel ecological conditions [28] associated with the appearance and disappearance of deep-freshwater lakes long the whole length of the EARS (Figure 1). The effects of the lake variability may have been enhanced at 1.8 Ma as key geological features that fragment the current lake Basins had yet to form; including the Barrier volcano separating Lake Turkana and the Suguta valley (∼1.4 Ma eastern side and ∼0.7 Ma western side) and the Emuruangogolak volcano (∼1.3 Ma), and Namarunu volcano (0.8 Ma) which separate Lake Baringo and the Suguta Valley [35]–[37], [44]. Hence during wet periods at about 1.8 Ma one huge lake may have extended perioidically from the Omo National Park in the north to just north of Lake Baringo in the south covering over 16,000 km^2^.

**Table 3:** The relationship between time and EARS lake coverage and encephalisation in hominins in Africa and Eurasia.

Model	AIC	adj r2	AIC	d.f.	AICchange	Coefficient	
Global	−537.84	0.90	Genus	1,181	55.7	0.09(0.04)	Homo
						−0.07(0.04)	Paranthropus
			Age		35.94	−0.13(0.02)	
			ODP 721		6.89	0.11(0.04)	
			ODP 967		3.42	0.07(0.03)	
			All lakes		1.63	−0.05(0.03)	
			ODP 659		0.16	−0.08(0.06)	
Africa	−134.2	0.88	Genus	1,46	24.24	0.07(0.06)	Homo
						−0.07(0.06)	Paranthropus
			Age		24.15	−0.19(0.03)	
			All lakes		10.47	−0.21(0.06)	
			ODP 967		1.37	−0.24(0.13)	
Asia	−439.44	0.79	Age	1,126	91.07	−0.16(0.01)	
			ODP 659		23.69	−0.33(0.02)	
			ODP 721		7.02	0.11(0.04)	
			All Lakes		0.42	0.04(0.02)	

All available paleoclimate databases were incorporated in the global models. Age was incorporated in the models to minimise the probability of spurious, non-causal temporal correlation between encephalisation and long-term paleoclimate trends. StepAIC was used to identify model with most support. Full model results are available in File S1.

The appearance of the *Homo erectus sensu lato* in Africa represents a fundamental turning point in hominin evolution. Not only was there a dramatic increase in brain size, but also in life history (shortened inter-birth intervals, delayed development), body size, shoulder morphology allowing throwing of projectiles [45], ecological flexibility [46] and social behaviour [47]. Some of these changes are consistent with a change in strategy towards flexibility and the ability to colonise novel environments. In contrast, the robust *Australopithecus sp.* adopted specialised habitat and dietary strategies [8]. Thus, two strategies arose during this period, one of increased flexibility and one of increased specialisation. There could be three evolutionary processes could explain this adaptive radiation of hominins: 1) the occupation of novel niches for species living in a highly productive but spatially constrained region when there are deep fresh water lakes in the EARS [46] and 2) the lakes themselves creating spatial structure producing population isolation and vicariance and 3) repeated periods of increased resource availability stimulated adaptation and radiation followed by periods of environmental stress when the lakes rapidly dried up imposing strong selection pressures [28]. Given that fossils from multiple hominin species, including *P. boisei, H. erectus spp.*, *H. habilis* and *H. rudolfensis,* have all been discovered at Koobi Fora and attributed to the period of maximal lake coverage (∼1.8–1.9 Ma), it may be possible that these hominins were sympatric and in competition [48]. Moreover, the *Paranthropus sp.* are associated with habitats that incorporate wetlands [8], [49], which would have been more available during periods of maximal lake coverage. It is interesting to note that hominin diversity drops from six down to only one during the period when deep-water lakes are in decline across the landscape (Figure 1) supporting the suggestion that competition was a major driver of hominin evolution. The Last Appearance Dates of the *Paranthropus sp.* is contemporary with the disappearance of deep-water lakes and an onset of an arid cycle.

## Conclusion

That the presence of Rift lakes is concurrent with major events in hominin evolution suggests that we must embrace a new perspective on how environmental conditions drove human evolution. Paleoclimate derived from stacked benthic foraminifera δ^18^O or aeolian dust records has been unable explain the occurrence of discrete evolutionary phases in the hominin fossil record [2]–[3], [15], the incorporation of the Rift lakes as both a climate indicator and landscape feature provides this missing environmental evidence. The unusual geology and climate of the East African Rift System introduced selective environmental pulses, which not only drove speciation but also subsequent dispersal events. However, our study suggests that this is not a simple relationship between climate variability and hominin evolution. As significant hominin brain expansion occurred at ∼1.8 Ma coeval with occurrence of massive ephemeral deep-water lakes, while subsequent expansions are associated with extreme dry periods. Hence, though the Pulse Climate Variability hypothesis is an interesting starting point for discussing early human evolution it may to simplistic to capture the whole range of evolutionary forcing occurring in East Africa in the Plio-Pleistocene. We may have to consider that climate was not always the underlying cause and that intrinsic social factors and intra-species competition may have play a significant role [50]. However, understanding the role of East African lakes does seem to be required to explain why and when hominin species arose and eventually left East Africa.

## Supporting Information

File S1 **Supporting figures and tables.** Table S1 Hominin diversity estimates based on First and Last Appearance dates (FAD and LAD). Table S2 Alternative scenarios for hominin migration events based on putative timings in the literature. Table S3 Best fit models for using regional and global dust flux records to predict occurrence of EARS lakes over the past 3 million years. Table S4 Best fit models for using regional and global dust flux records to predict occurrence of EARS lakes from 350 Ka to 2.6 Ma. Table S5 Models for hominin diversity. Table S6 Stepwise AIC model selection for species turnover (net change in species richness). Table S7 Migration models for three migration scenarios. Table S8 Best fit models for brain capacity over all hominins. Table S9 Regional brain capacity models Figure S1 Comparison of the probability of a dispersal or migration event out of East Africa with the Lake variability index.
(DOC)
