# Supplementary material for: Early Human Speciation, Brain Expansion and Dispersal Influenced by African Climate Pulses
Source: PLoS One. 2013 Oct 16;8(10):e76750. doi: 10.1371/journal.pone.0076750 (PMC3797764; doi:10.1371/journal.pone.0076750)
Supplement: File S1 — Supporting figures and tables. Table S1 Hominin diversity estimates based on First and Last Appearance dates (FAD and LAD). Table S2 Alternative scenarios for hominin migration events based on putative timings in the literature. Table S3 Best fit models for using regional and global dust flux records to predict occurrence of EARS lakes over the past 3 million years. Table S4 Best fit models for using regional and global dust flux records to predict occurrence of EARS lakes from 350 Ka to 2.6 Ma. Table S5 Models for hominin diversity. Table S6 Stepwise AIC model selection for species turnover (net change in species richness). Table S7 Migration models for three migration scenarios. Table S8 Best fit models for brain capacity over all hominins. Table S9 Regional brain capacity models Figure S1 Comparison of the probability of a dispersal or migration event out of East Africa with the Lake variability index. (DOC) [file pone.0076750.s001.doc]

**Supporting Information: Early human speciation, brain expansion and dispersal influenced by African climate pulses**

Susanne Shultz^1^ and Mark Maslin^2*^

1. Faculty of Life Sciences, The University of Manchester, Manchester, UK

2. Department of Geography, University College London, London, UK

* corresponding author m.maslin@ucl.ac.uk

*Lake variability as a predictor of local climate*

The geologic record of East African rift (EAR) lakes indicate a pattern of temporal flux and the lakes, therefore, have been suggested as a proxy for local paleoclimatic conditions. However, there is still some disagreement as to what extent these lakes truly reflect historic patterns of climate conditions or are the result of tectonic activity. Trauth et al. [1] demonstrated that trends in dust records from the Arabian Sea, West Africa and the Mediterranean Sea are not correlated and are picking up different underlying climate responses in the regions of dust origin (or variation in deposition patterns). The dust records surrounding Northern, Eastern and Western Africa should together provide a picture of regional climate variation. There is also a contention that globally integrated palaeoclimate records such as the stacked benthic foraminifera δ^18^O, representing a combination of global ice volume and bottom water temperatures, may be a more appropriate comparison with hominin changes [2-4]. We evaluated whether the EAR lake occurrence can be predicted by variation in accepted African paleoclimate records based on aeolian terrigenous flux in marine cores and global stacked benthic foraminifera δ^18^O records [5].

Lake variability index was calculated by collating the published geological evidence for the appearance of either deep ephemeral or shallow alkaline lakes in seven major Basins [6-9]. The Deep Lake index only includes evidence for large deep ephemeral lakes, whereas the All Lake index includes evidence for any standing water in the lake basins. We present the results for the All Lake index in the main manuscript, as the models suggest this measure picks up a finer scale picture of climate variability (i.e. incorporates more paleoclimate indicators). The lakes include the Olduvai Basin (Tanzania), Magadi-Natron-Olorgesailie Basin (N. Tanzania and S. Kenya), Central Kenya Rift Basins (Kenya), Baringo-Bogoria Basin (Kenya), Omo-Turkana-Suguta Basin (N. Kenya), Ethiopian Rift (South and Central Ethiopia) and Afar Basin (N. Ethiopia). The index was normalised by dividing by 7 to produce a range from 0 to 1. Mean and standard deviation for each 100 kyr block prior to each specimen date were calculated to provide an estimate of change and variability for other paleoclimate records (Mediterranean Sea [10], Arabian Sea [11] and West African [12] and stacked benthic foraminifera δ^18^O records [5]). The paleoclimate databases were aggregated by taking the mean of records for each 50 kyr period.

*Hominin evolution*

East African hominin diversity at each 100 kyr interval were estimated using first (FAD) and last appearance dates (FAD) as provided in Reed and Fish [13], with date changes provided by Joordens et al [14], McDougall et al [15] and Leakey et al [16]. A diversity index was calculated by summing the number of extant hominin species over every 100 kyr block (Table S1). We aggregated the above paleoclimate records into 100 kyr blocks to align with the hominin diversity index. We did not use finer resolution blocks for these analyses as the fossil dating of first and last appearance is subject to significant error. There is extensive ongoing discussion about whether *Homo erectus* and *H. ergaster* should be treated as the same species [17-18]. We treated Homo erectus as a superspecies, but included continent as the key distinguishing feature in the brain size models to identify any regional differences in evolutionary trends. Hominin migration dates were estimated by FAD of hominin specimens outside of EARS [19-21]. In order to reflect the different migration time-points that have been advocated in the literature, we used three plausible migration scenarios (Table S2). Although first appearance times are likely to be an underestimate of taxa age and migration dates, first appearance dates are the best available evidence for speciation and migration events. Hominin specimen dates and brain size estimates were taken from Shultz et al [22], with a few deposition dates for East African sites modified using the recently published revised dates from McDougall et al [15]; Joordens et al [14]. For the brain size analyses, specimen dates were matched with paleoclimate for each associated 50 kyr period.

*Statisical analyses*

We then used a stepAIC approach to identify the combination(s) of paleoclimate records which best predict the lake index. The change in AIC in table 1 and 2 represent the change in the model AIC when each successive term is dropped. We finally included the variables from the model with the lowest AIC in a linear model to evaluate model adjusted r^2^. For migration and species turnover analyses, where the response variable was binary, we used a binomial error distribution with log link and do not report r^2^. For all analyses, we checked for co-linearity using a variance inflation factor; any predictors with vif higher than 5 were dropped from the model (vif, package HH in R).

To determine whether the lake index is correlated with either regional specific or global stacked ocean records, we identified which, if any, paleoclimate dust records are associated variability in lake occurrence in the EARS. The three aggregated 50 kyr regional dust records and benthic foraminifera δ^18^O record were normalised between 0 and 1 to ensure equal variances for each time series. We also used the average path length network measure developed by Donges et al [23] to identify periods of rapid climate change. For this analysis, data were truncated to the most limited set of data (i.e. 0-3 Ma for the dust records and 350 ka - 2.6 Ma for the analyses including the measures of average path length). We also ran models without these variables covering a longer time period of 0-3 Ma.

In order to identify paleoclimate indicators associated with diversity and migration events, we used a GLM together with stepwise model selection (stepAIC function in R package MASS). As diversity indices are likely to be impacted by temporal autocorrelation (i.e. time periods with higher diversity are more likely to see a speciation or events), we additionally ran models that used net change as a dependent variable and species diversity as a model factor.

Brain size was evaluated initially using a general linear model over all available specimens, with specimen age and paleoclimate data incorporated as covariates and genus and continent added as main factors. Age was included to remove underlying step changes in brain size associated with shifts in the paleoclimate record as opposed to simple correlated temporal trends. The data were then analysed separately for each continent to highlight the potential different processes.

*Results*

The best predictors for the occurrence of all EARS lakes (both deep and shallow) were the global stacked data and all three regional records, clearly showing that the lakes represent primarily a regional climate signal and not local tectonics (Table S3). The Mediterranean and the West African dust records suggest that EARS lakes are more abundant during wetter periods. The Arabian Sea dust record is positively correlated with the lake index, suggesting that lakes are more abundant during periods of higher variability in Arabian dust deposition, as suggested by Deino et al [24]; Kingston et al. [25], and Trauth et al. [8]. Likewise, when we evaluate only the deep lakes, the best indicators of lake occurrence is the West African dust record (wet periods associated with more lakes) and higher variability in Mediterranean dust deposition.

When the indicator of climate turnover, APL (Average Path Length), were incorporated into the analyses, both the Mediterranean and West African APLs records provided the most predictive power (Table S4). Periods of rapid climate change, together with periods of low terrigenous flux, are associated with increased lake abundance. This strongly suggests that lakes in the EARS were a product of pulses of wet weather, which are fundamentally different than the underlying long-term trends, or orbital cycles, in the global record.

For both species diversity (Table S5) and dispersal events (Table S7, Figure S1), the best fit models included only a measure of lake presence. However, for species turnover (Table S6), the best model additionally included the Mediterranean terrigenous flux index and the hominin species richness. This model, unlike the species diversity model, had no evidence of temporal autocorrelation.

Post 1.8-2.0 Ma bigger brains are associated with fewer lakes and more dust off West Africa, indicating periods of brain expansion are associated with periods of dry climate in Africa (Table S8-S9). Conversely, brain size increase is associated with wet periods in the Mediterranean dust flux though there is a much stronger relationship with hominins in Eurasia than in Africa. This suggests that post 1.8 Ma brain expansion is driven by arid conditions in West and East Africa, but the migration of those new species is driven by wet period in East Africa and the Mediterranean.

Reference

1 Trauth MH, Larrasoaña JC, Mudelsee M (2009) Trends, rhythms and events in Plio-Pleistocene African climate. Quaternary Science Reviews 28: 399-411, doi:10.1016/j.quascirev.2008.11.003.

2 Grove M (2011) Change and variability in Plio-Pleistocene climates: modelling the hominin response. Journal of Archaeological Science 38: 3038-3047, doi:10.1016/j.jas.2011.07.002.

3 Grove M (2011) Speciation, diversity, and Mode 1 technologies: The impact of variability selection. Journal of Human Evolution 61: 306-319, doi:10.1016/j.jhevol.2011.04.005.

4 Grove M (2012) Amplitudes of orbitally induced climatic cycles and patterns of hominin speciation. Journal of Archaeological Science 39: 3085-3094, doi:10.1016/j.jas.2012.04.023.

5 Lisiecki LE, Raymo ME (2005) A Pliocene-Pleistocene stack of 57 globally distributed benthic delta18O records. Paleoceanography 20: PA1003, doi:10.1029/2004pa001071.

6 Maslin MA, Trauth MH (2009) Plio-Pleistocene East African Pulsed Climate Variability and its influence on early human evolution, In "The First Humans - Origins of the Genus Homo" (eds FE Grine, RE Leakey, JG Fleagle) 151- 158.

7 Trauth MH, Maslin MA, Deino A, Strecker M (2005) Late Cenozoic moisture history of East Africa. Science 309: 2051-2053.

8 Trauth MH, Maslin M, Deino A, Strecker MR, Bergner AGN, Duhnforth M (2007) High and low latitude forcing of Plio-Pleistocene East African climate and human evolution. Journal of Human Evolution 53: 475-486.

9 Trauth MH, Maslin MA, Bergner A, Deino A, Junginger A, et al. (2010) Human evolution in a variable environment: the amplifier lakes of Eastern Africa. Quaternary Science Reviews 29: 2981-2988.

10 Larrasoaña JC, Roberts AP, Rohling EJ, Winklhofer M, Wehausen R (2003) Three million years of monsoon variability over the northern Sahara. Climate Dynamics 21: 689-698.

11 deMenocal PB (1995) Plio-Pleistocene African climate. Science, 270: 53-59, doi:10.1126/science.270.5233.53.

12 Tiedemann R, Sarnthein M, Shackleton NJ (1994) Astronomic Timescale for the Pliocene Atlantic delta18O and Dust Flux Records of Ocean Drilling Program Site 659. Paleoceanography 9: 619-638, doi:10.1029/94pa00208.

13 Reed KE, Fish JL (2005) Tropical and temperate seasonal influences on human evolution. In Seasonality in Primates: Studies of Living and Extinct Human and Non-Human Primates. (Brockman DK, van Schaik CP editors) Cambridge University Press: 491-520.

14 Joordens JCA, Vonhof HB, Feibel CS, Lourens LJ, Dupont-Nivet G, et al. (2011) An astronomically-tuned climate framework for hominins in the Turkana Basin. Earth and Planetary Science Letters 307: 1-8.

15 McDougall I, Brown, FH, Vasconcelos PM, Cohen BE, Thiede DS, et al. (2012) New single crystal 40Ar/39Ar ages improve time scale for deposition of the Omo Group, Omo‚Turkana Basin, East Africa. Journal of the Geological Society 169: 213-226, doi:10.1144/0016-76492010-188.

16 Leakey MG, Spoor F, Dean MC, Feibel CS, Anton S et al. (2012) New fossils from Koobi Fora in northern Kenya confirm taxonomic diversity in early Homo. Nature 488: 201-204.

17 Antón SC (2003) Natural history of Homo erectus. American Journal of Physical Anthropology 122: 126-170, doi:10.1002/ajpa.10399.

18 Baab KL, McNulty KP (2009) Size, shape, and asymmetry in fossil hominins: the status of the LB1 cranium based on 3D morphometric analyses. Journal of Human Evolution 57: 608-622, doi:10.1016/j.jhevol.2008.08.011.

19 O'Regan HJ, Turner A, Bishop LC, Elton S, Lamb AL (2011) Hominins without fellow travellers? First appearances and inferred dispersals of Afro-Eurasian large-mammals in the Plio-Pleistocene. Quaternary Science Reviews 30: 1343-1352, doi:10.1016/j.quascirev.2009.11.028.

20 Hughes JK, Haywood A, Mithen SJ, Sellwood BW, Valdes PJ (2007) Investigating early hominin dispersal patterns: developing a framework for climate data integration. Journal of Human Evolution 53, 465-474, doi:10.1016/j.jhevol.2006.12.011.

21 Pares JM, Duval M, Arnold LJ (2013) New views on an old move: Hominin migration into Eurasia. Quaternary International, 295: 5-12, doi:10.1016/j.quaint.2011.12.015.

22 Shultz S, Nelson, Dunbar RIM (2012) Hominin cognitive evolution: identifying patterns and processes in the fossil and archaeological record. Philosophical Transactions of the Royal Society B: Biological Sciences 367: 2130-2140, doi:10.1098/rstb.2012.0115.

23 Donges JF, Donner RV, Trauth MH, Marwan N, Schellnhuber H-J (2011) Nonlinear detection of paleoclimate-variability transitions possibly related to human evolution. Proceedings of the National Academy of Sciences of the United States of America 108: 20422–20427, doi:10.1073/pnas.1117052108.

24 Deino AL, Kingston JD, Glen JM, Edgar RK, Hill A (2006) Precessional forcing of lacustrine sedimentation in the late Cenozoic Chemeron Basin, Central Kenya Rift, and calibration of the Gauss/Matuyama boundary. Earth and Planetary Science Letters 247: 41-60.

25 Kingston J, Deino A, Edgar R, Hill A (2007) Astronomically forced climate change in the Kenyan Rift Valley 2.7 - 2.55 Ma: Implications for the evolution of early hominin ecosystems. Journal of Human Evolution 53: 487-503.

Table S1. Hominin diversity estimates based on First and Last Appearance dates (FAD and LAD) as described in the text.

Table S2. Alternative scenarios for hominin migration events based on putative timings in the literature [19-21]. Filled time blocks represent periods with probable migration events. We ran each scenario through a model selection procedure.

|  | Migration Scenario | | |
| --- | --- | --- | --- |
| Time Kya | A | B | C |
| 3000 |  |  |  |
| 2900 |  |  |  |
| 2800 |  |  |  |
| 2700 |  |  |  |
| 2600 |  |  |  |
| 2500 |  |  |  |
| 2400 |  |  |  |
| 2300 |  |  |  |
| 2200 |  |  |  |
| 2100 |  |  |  |
| 2000 |  |  |  |
| 1900 |  |  |  |
| 1800 | 1 |  |  |
| 1700 | 1 | 1 | 1 |
| 1600 |  |  |  |
| 1500 |  |  |  |
| 1400 |  | 1 |  |
| 1300 |  |  |  |
| 1200 |  |  |  |
| 1100 |  |  |  |
| 1000 |  |  |  |
| 900 |  |  |  |
| 800 | 1 | 1 | 1 |
| 700 |  |  |  |
| 600 |  |  |  |
| 500 |  |  |  |
| 400 |  |  |  |
| 300 |  |  |  |
| 200 |  |  |  |
| 100 | 1 | 1 | 1 |
| 0 | 1 | 1 | 1 |

Table S3. Best fit models for using regional and global dust flux records to predict occurrence of EARS lakes over the past 3 million years. Ocean drilling program site ODP 721/722 from the Arabian Sea, site ODP 659 from off the coast of West Africa and ODP 967 from the Mediterranean Sea. AIC change represents the increase in the model AIC after sequentially dropping each retained term in the best fir model. The global model contains all predictor variables. A linear model was run on the model with the lowest AIC to extract parameter coefficients and significance values (for interpretation). Adjusted r^2^ values are reported for the model with the lowest AIC values as an indication of variance explained by the model. Parameter coefficients are presented with standard errors in parentheses.

|  | Model | AIC | Predictors | d.f. | AIC change | Coefficients | adj r2 |
| --- | --- | --- | --- | --- | --- | --- | --- |
| ALL LAKES | Global | -18.99 | ODP721/722 | 1,56 | 2.34 | 0.39 (0.19) | 0.16 |
|  |  |  | ODP967 |  | 3.13 | -0.41 (0.18) |  |
|  |  |  | δ^18^O Stack |  | 3.78 | 1.32 (0.56) |  |
|  |  |  | ODP659 |  | 4.09 | -0.55 (0.22) |  |
| DEEP LAKES | 1 | -49.86 | ODP659 | 1,58 | 3.62 | -0.41 (0.17) | 0.19 |
|  |  |  | ODP721/722 |  | 12.97 | 0.51 (0.13) |  |
|  | 2 | -47.88 | ODP967 |  | -1.98 |  |  |
|  |  |  | ODP659 |  | 3.41 |  |  |
|  |  |  | ODP721/722 |  | 10.13 |  |  |
|  | Global | -45.92 | δ^18^O Stack |  | -1.96 |  |  |
|  |  |  | ODP967 |  | -1.95 |  |  |
|  |  |  | ODP659 |  | 3.41 |  |  |
|  |  |  | ODP721/722 |  | 8.7 |  |  |

Table S4. Best fit models for using regional and global dust flux records to predict occurrence of EARS lakes from 350 ka to 2.6 Ma. Ocean drilling program site ODP 721/722 from the Arabian Sea, site ODP 659 from off the coast of West Africa and ODP 967 from the Mediterranean Sea. AIC change represents the increase in the model AIC after sequentially dropping each retained term in the best fir model. The global model contains all predictor variables. A linear model was run on the model with the lowest AIC to extract parameter coefficients and significance values (for interpretation). Adjusted r^2^ values are reported for the model with the lowest AIC values as an indication of variance explained by the model. APL predictors are the average path lengths from Donges et al 2011: high values are associated with periods of rapid climate change. Alternative models selected by stepAIC function; models with <2 AIC difference from sequential best fit models included in stepAIC output.

|  | Model | AIC | Predictors | d.f. | AIC change | adj r2 | coefficient |
| --- | --- | --- | --- | --- | --- | --- | --- |
| ALL LAKES | 1 | -37.48 | ODP 721/722 | 1,39 | 0.35 | 0.49 | 0.29 (0.20) |
|  |  |  | ODP 659 |  | 3.38 |  | -0.24 (0.11) |
|  |  |  | ODP 967 |  | 3.79 |  | -0.53 (0.22) |
|  |  |  | δ^18^O stack |  | 5.39 |  | 1.64 (0.63) |
|  |  |  | APL ODP 659 |  | 6.66 |  | 0.09 (0.03) |
|  |  |  | APL ODP 967 |  | 7.58 |  | 0.07 (0.02) |
|  | Global | -35.84 | APL ODP 721/722 |  | -1.64 |  |  |
|  |  |  | ODP 721/722 |  | 0.17 |  |  |
|  |  |  | ODP 659 |  | 3.14 |  |  |
|  |  |  | ODP 967 |  | 3.97 |  |  |
|  |  |  | δ^18^O stack |  | 5.73 |  |  |
|  |  |  | APL ODP 659 |  | 6.66 |  |  |
|  |  |  | APL ODP 967 |  | 7.34 |  |  |
| DEEP LAKES ONLY | 1 | -41.78 | ODP 967 | 1,41 | 0.7 | 0.19 | -0.26 (0.17) |
|  |  |  | ODP 721/722 |  | 1.83 |  | 0.35 (0.18) |
|  |  |  | APL ODP967 |  | 2.05 |  | 0.043 (0.02) |
|  |  |  | ODP659 |  | 2.46 |  | -0.21 (0.10) |
|  | 2 | -40.92 | APL ODP721/722 |  | -0.86 |  |  |
|  |  |  | ODP 967 |  | 1.67 |  |  |
|  |  |  | APL ODP967 |  | 1.82 |  |  |
|  |  |  | ODP 721/722 |  | 1.89 |  |  |
|  |  |  | ODP 659 |  | 2.06 |  |  |
|  | 3 | -39.7 | APL ODP659 |  | -1.22 |  |  |
|  |  |  | APL ODP 721/722 |  | -0.95 |  |  |
|  |  |  | ODP 967 |  | -0.32 |  |  |
|  |  |  | ODP 659 |  | 0.96 |  |  |
|  |  |  | APL ODP 967 |  | 1.66 |  |  |
|  |  |  | ODP721/722 |  | 1.76 |  |  |
|  | Global | -38.57 | δ^18^O stack |  | -1.13 |  |  |
|  |  |  | APL ODP659 |  | -0.97 |  |  |
|  |  |  | APL ODP 721/722 |  | -0.49 |  |  |
|  |  |  | ODP 721/722 |  | 0.16 |  |  |
|  |  |  | ODP 967 |  | 0.55 |  |  |
|  |  |  | ODP 659 |  | 0.89 |  |  |
|  |  |  | APL ODP967 |  | 1.58 |  |  |

Table S5. Models for hominin diversity. Global model includes all factors entered into original model. Alternative models selected by stepAIC function; models with <2 AIC difference from sequential best fit models included in stepAIC output. Species turnover is estimated by species richness in consecutive time periods (T*_i_*-T*_i-1_*).

| Model | AIC | adj r2 | Predictor | AIC change | Coefficient |
| --- | --- | --- | --- | --- | --- |
| 1 | 96.501 | 0.49 | ODP 967 | 6.534 | -2.8 (0.95) |
|  |  |  | All lakes | 19.508 | 5.9 (1.21) |
| 2 | 97.71 |  | ODP 659 | -1.209 |  |
|  |  |  | ODP 967 | 6.227 |  |
|  |  |  | All lakes | 19.92 |  |
| 3 | 99.663 |  | δ^18^O | -1.953 |  |
|  |  |  | ODP 659 | -1.502 |  |
|  |  |  | ODP 967 | 4.674 |  |
|  |  |  | All lakes | 18.285 |  |
| Global | 101.628 |  | ODP721/722 | -1.965 |  |
|  |  |  | δ^18^O | -1.947 |  |
|  |  |  | ODP 659 | -1.485 |  |
|  |  |  | ODP 967 | 2.567 |  |
|  |  |  | All lakes | 18.035 |  |

Table S6. Stepwise AIC model selection for species turnover (net change in species richness).

| Model | AIC | adj r2 | Predictor | AIC change | Coefficient |
| --- | --- | --- | --- | --- | --- |
| 1 | 79.11 | 0.18 | intercept | -0.003 | -1.13 (0.48) |
|  |  |  | ODP967 | 2.551 | 2.10 (1.01) |
|  |  |  | Deep lakes | 3.986 | -2.69 (1.12) |
|  |  |  | African diversity | 6.738 | 0.39 (0.13) |
| 2 | 80.07 |  | ODP659 | -0.963 |  |
|  |  |  | ODP967 | -0.26 |  |
|  |  |  | intercept | -0.001 |  |
|  |  |  | Deep lakes | 2.316 |  |
|  |  |  | African diversity | 6.403 |  |
| 3 | 81.1 |  | global O18 | -1.031 |  |
|  |  |  | ODP659 | -0.233 |  |
|  |  |  | intercept | 0.003 |  |
|  |  |  | ODP967 | 0.57 |  |
|  |  |  | Deep lakes | 1.863 |  |
|  |  |  | African diversity | 6.026 |  |
| Global | 82.88 |  | ODP721 | -1.777 |  |
|  |  |  | global O18 | -0.876 |  |
|  |  |  | ODP659 | -0.428 |  |
|  |  |  | intercept | -0.005 |  |
|  |  |  | ODP967 | 0.053 |  |
|  |  |  | Deep lakes | 1.907 |  |
|  |  |  | African diversity | 6.193 |  |

Table S7. Migration models for three migration scenarios. R^2^ is not reported as the models assume a binomial error with log link.

| Scenario |  | AIC | Predictors | AIC change | Coefficients |
| --- | --- | --- | --- | --- | --- |
| A | 1 | 16.04 | Intercept | 0.00 | -5.50 (2.15) |
|  |  |  | Deep Lakes | 13.35 | 17.14 (7.64) |
|  | 2 | 16.49 | Global O18 | -0.45 |  |
|  |  |  | intercept | 0.00 |  |
|  |  |  | Deep Lakes | 12.45 |  |
|  | 3 | 18.31 | ODP967 | -1.82 |  |
|  |  |  | Global O18 | -1.79 |  |
|  |  |  | Intercept | 0.00 |  |
|  |  |  | Deep Lakes | 10.54 |  |
|  | Global | 20.12 | ODP659 | -1.81 |  |
|  |  |  | ODP967 | -1.70 |  |
|  |  |  | Global O18 | -1.65 |  |
|  |  |  | Intercept | 0.00 |  |
|  |  |  | Deep Lakes | 7.48 |  |
| B | 1 | 26.02 | Intercept | 0.11 | -2.86 (0.88) |
|  |  |  | Deep Lakes | 3.30 | 6.30 (3.12) |
|  | 2 | 26.13 | Intercept | 0.00 |  |
|  |  |  | Deep Lakes | 0.01 |  |
|  |  |  | ODP967 | 0.07 |  |
|  | 3 | 28.12 | ODP659 | -1.99 |  |
|  |  |  | ODP967 | -0.65 |  |
|  |  |  | Deep Lakes | -0.41 |  |
|  |  |  | Intercept | 0.00 |  |
|  | Global | 30.12 | Global O18 | -2.00 |  |
|  |  |  | ODP659 | -2.00 |  |
|  |  |  | ODP967 | -1.25 |  |
|  |  |  | Deep Lakes | -0.45 |  |
|  |  |  | Intercept | 0.00 |  |
| C | 1 | 18.23 | Intercept | 0.00 | -84.69 (43.9) |
|  |  |  | ODP659 | 0.75 | -33.07 (23.67) |
|  |  |  | Deep Lakes | 2.62 | 12.8 (7.69) |
|  |  |  | Global O18 | 7.23 | 106.09 (58.20) |
|  | 2 | 18.94 | Intercept | 0.05 |  |
|  |  |  | Global O18 | 5.29 |  |
|  |  |  | Deep Lakes | 4.84 |  |
|  | Global | 20.21 | ODP967 | -1.89 |  |
|  |  |  | Intercept | 0.09 |  |
|  |  |  | Deep Lakes | 0.63 |  |
|  |  |  | ODP659 | 0.86 |  |
|  |  |  | Global O18 | 2.75 |  |

Table S8. Best fit models for brain capacity over all hominins. Ocean drilling program site ODP 721/722 from the Arabian Sea, site ODP 659 from off the coast of West Africa and ODP 967 from the Mediterranean Sea. Standard error of the mean presented with coefficients. Models are those retained using a stepAIC model selection. N=181. Global model includes all factors entered into original model. Alternative models selected by stepAIC function; models with <2 AIC difference from sequential best fit models included in stepAIC output.

| Model | AIC | adj r2 | AIC | AIC change | Coefficient |  |
| --- | --- | --- | --- | --- | --- | --- |
| 1 | -537.84 | 0.90 | ODP 659 | 0.16 | -0.08 (0.06) |  |
|  |  |  | All lakes | 1.63 | -0.05 (0.03) |  |
|  |  |  | ODP 967 | 3.42 | 0.07 (0.03) |  |
|  |  |  | ODP 721 | 6.89 | 0.11 (0.04) |  |
|  |  |  | Age | 35.94 | -0.13 (0.02) |  |
|  |  |  | Genus | 55.7 | 0.09 (0.04) | Homo |
|  |  |  |  |  | -0.07 (0.04) | Paranthropus |
| 2 | -536.83 |  | δ^18^O | -1.01 |  |  |
|  |  |  | ODP 659 | 0.27 |  |  |
|  |  |  | All lakes | 2.2 |  |  |
|  |  |  | ODP 967 | 4.31 |  |  |
|  |  |  | ODP 721 | 7.81 |  |  |
|  |  |  | Age | 36.8 |  |  |
|  |  |  | Genus | 55.25 |  |  |
| Global | -535.25 |  | Continent | -1.58 |  |  |
|  |  |  | δ^18^O | -1.27 |  |  |
|  |  |  | ODP 659 | 0.02 |  |  |
|  |  |  | All lakes | 2.46 |  |  |
|  |  |  | ODP 967 | 3.9 |  |  |
|  |  |  | ODP 721 | 8.2 |  |  |
|  |  |  | Age | 30.29 |  |  |
|  |  |  | Genus | 49.95 |  |  |

Table S9. Regional brain capacity models. N = 51 Africa and N = 129 Asia. Global model includes all factors entered into original model. Alternative models selected by stepAIC function; models with <2 AIC difference from sequential best fit models included in stepAIC output.

| Region | Model | AIC | adj r2 | Predictor | AIC change | Coefficient |  |
| --- | --- | --- | --- | --- | --- | --- | --- |
| Africa | 1 | -134.2 | 0.88 | ODP 967 | 1.37 | -0.24 (0.13) |  |
|  |  |  |  | All lakes | 10.47 | -0.21 (0.06) |  |
|  |  |  |  | Age | 24.15 | -0.19 (0.03) |  |
|  |  |  |  | genus | 24.24 | 0.07 (0.06) | Homo |
|  |  |  |  |  |  | -0.07 (0.06) | Paranthropus |
|  | 2 | -132.75 |  | δ^18^O | -1.45 |  |  |
|  |  |  |  | ODP 967 | 1.63 |  |  |
|  |  |  |  | All lakes | 9.7 |  |  |
|  |  |  |  | Age | 18.52 |  |  |
|  |  |  |  | genus | 23.41 |  |  |
|  | 3 | -130.88 |  | ODP 659 | -1.87 |  |  |
|  |  |  |  | δ^18^O | -1.58 |  |  |
|  |  |  |  | ODP 967 | 1.73 |  |  |
|  |  |  |  | All lakes | 9.79 |  |  |
|  |  |  |  | Age | 15.43 |  |  |
|  |  |  |  | genus | 22.5 |  |  |
|  | Global | -128.91 |  | ODP 721 | -1.97 |  |  |
|  |  |  |  | ODP 659 | -1.91 |  |  |
|  |  |  |  | δ^18^O | -1.57 |  |  |
|  |  |  |  | ODP 967 | -0.32 |  |  |
|  |  |  |  | Age | 6.21 |  |  |
|  |  |  |  | All lakes | 9.14 |  |  |
|  |  |  |  | Genus | 22.52 |  |  |
| Asia | 1 | -439.44 | 0.79 | All Lakes | 0.42 | 0.04(0.02) |  |
|  |  |  |  | ODP 721 | 7.02 | 0.11 (0.04) |  |
|  |  |  |  | ODP 659 | 23.69 | -0.33 (0.02) |  |
|  |  |  |  | Age | 91.07 | -0.16 (0.01) |  |
|  | 2 | -437.8 |  | δ^18^O | -1.64 |  |  |
|  |  |  |  | All Lakes | 0.12 |  |  |
|  |  |  |  | ODP 721 | 6.77 |  |  |
|  |  |  |  | ODP 659 | 22.96 |  |  |
|  |  |  |  | Age | 63 |  |  |
|  | Global | -435.97 |  | ODP 967 | -1.83 |  |  |
|  |  |  |  | δ^18^O | -1.47 |  |  |
|  |  |  |  | All lakes | 0.13 |  |  |
|  |  |  |  | ODP 721 | 6.9 |  |  |
|  |  |  |  | ODP 659 | 15.85 |  |  |
|  |  |  |  | Age | 47.05 |  |  |

Figure S1.


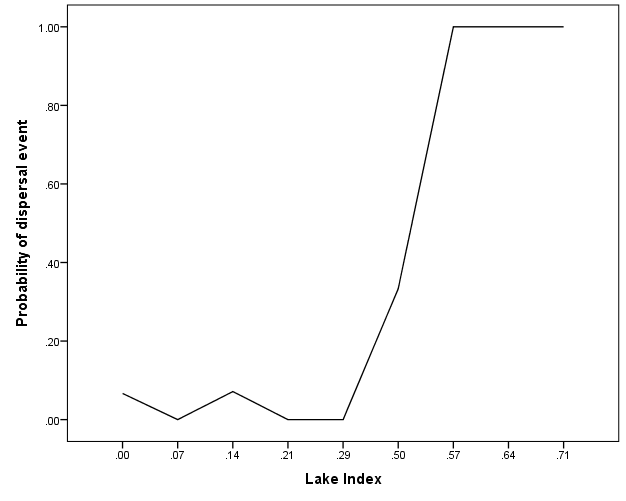


Figure S1 Comparison of the probability of a dispersal or migration event out of East Africa with the Lake variability index. Threshold clearly shows that once a substantial number of Basins are full of lakes hominins are pushed out of East Africa.
